# Uncovering communication strategies used in language‐discordant consultations with people who are migrants: Qualitative interviews with healthcare providers

**DOI:** 10.1111/hex.13949

**Published:** 2023-12-25

**Authors:** Brittany M. C. Chan, Jeanine Suurmond, Julia C. M. van Weert, Barbara C. Schouten

**Affiliations:** ^1^ Department of Communication Science, Amsterdam School of Communication Science (ASCoR) University of Amsterdam Amsterdam The Netherlands; ^2^ Department of Public and Occupational Health, Amsterdam UMC University of Amsterdam Amsterdam The Netherlands

**Keywords:** communication strategies, healthcare providers, interpreters, language‐discordant consultations, migrant patients

## Abstract

**Background:**

Global migration has led to a sharp increase in the number of language‐discordant consultations (LDCs) in healthcare. Evidence on how healthcare providers (HCPs) meet migrant patients' needs while mitigating language barriers is lacking.

**Design:**

Using purposive and snowball sampling, we recruited twenty‐seven Dutch HCPs (*M*_age_ = 45.07, SD = 11.46) and conducted semi‐structured interviews to collect qualitative, open‐ended data for identifying the communication strategies used with migrant patients in LDCs. We analysed the transcripts using deductive and inductive approaches (e.g., constant comparative method from Grounded Theory). Final pattern codes (i.e., key themes) were discussed among the research team until mutual agreement had been achieved.

**Results:**

Five key themes emerged from the analyses: HCPs often ‘got‐by’ with (1) instrumental and (2) affective communication strategies used in language‐concordant consultations to start medical consultations. When some instrumental communication strategies were deemed ineffective (e.g., lingua franca, gesturing, etc.) to bridge language barriers, HCPs turned to (3) incorporating digital tools (e.g., Google Translate). When HCPs were unable to communicate with migrant patients at all, (4) informal, ad‐hoc and professional interpreters were involved. Finally, HCPs often (5) involved additional support to engage migrant patients to engage in treatment‐related behaviours.

**Discussion and Conclusions:**

Our results highlight the importance of raising awareness among HCPs about using various combinations of different strategies. The development of a guideline indicating the optimal combination of communication strategies for different medical consultation goals may be useful in reshaping the current communication behaviour of HCPs in LDCs.

**Patient or Public Contribution:**

HCPs were the study population involved in this qualitative study. Refugee health advisors, general practitioners and linguistic specialists (i.e., members of the *Right2Health* consortium) with experience with the Dutch healthcare system were involved throughout the development of this research. This includes a review of the research question, participant information sheet and interview topic guide as well as providing interpretations of the data and feedback to this manuscript.

## 1 INTRODUCTION

In the Netherlands, approximately 10% of the migrant population does not speak Dutch at home.^1^ When healthcare providers (HCPs) and migrant patients do not sufficiently share a common language, communication quality is negatively affected. Compared to language‐concordant consultations, language‐discordant consultations (LDCs) often result in HCPs' incomplete comprehension of patients' medical situations, incorrect and delayed diagnoses and poorer patient outcomes, such as low‐quality treatment, poor patient safety and patient dissatisfaction.^2^, ^3^, ^4^ Without proper ways of mitigating language barriers, medical communications in LDCs are likely to be ineffective, thereby negatively impacting the quality of care migrant patients receive, creating a burden for society due to additional costs incurred^5^ and hampering the migrant population's access to healthcare.^6^, ^7^, ^8^

The *Six Function Model of Medical Communication* ^9^ outlines six goals: (1) *fostering a relationship*, (2) *gathering information*, (3) *providing information*, (4) *decision‐making*, (5) *enabling disease and treatment‐related behaviours* and (6) *responding to emotions* that guide HCPs in meeting patients' *cognitive* and *affective* needs intertwiningly to reach effective medical communication. When HCPs successfully meet these six goals, they likely fulfil patients' needs; they can help patients understand their illnesses and what actions need to be taken (i.e., cognitive needs), as well as ensure patients feel understood, supported and respected (i.e., affective needs). Establishing *if* each goal is met effectively is determined by observable consequences resulting from the *instrumental* and *affective communication strategies* used, that is, behaviours displayed by HCPs to convey information (e.g., repeating/rephrasing medical jargons) and build a connection (e.g., engaging in small talks) (see De Haes & Bensing et al.^9^, ^10^, ^11^, ^12^, ^13^ for other communication strategies). Using these communication strategies as stand‐alone strategies in LDCs is insufficient though. For instance, simply repeating and rephrasing information in HCPs' language will not increase low language proficient (LLP) migrant patients' understanding, as these instrumental communication strategies do not combat the main barrier—the language barrier.^4^, ^14^ As the current framework was developed for language‐concordant consultations, research on the communication strategies HCPs use in LDCs to achieve the six goals of LLP migrant patients' needs and mitigate language barriers is lacking. This is problematic as HCPs currently lack clear guidance on what combinations of communication strategies they can use to effectively communicate in LDCs and improve the quality of care for migrant patients.^15^

For instance, to mitigate language barriers in LDCs, HCPs can work with interpreters (professional, ad‐hoc and informal) and use digital tools.^16^, ^17^ Amongst the three types of interpreters, professional interpreters provide the most accurate medical interpretations, help safeguard migrant patients' safety and raise the overall quality of communication for patients in LDCs.^18^ Nonetheless, HCPs underuse professional interpretation services because of shortage of time, lack of availability and fear of upsetting the patient if they have arranged their own (informal) interpreter.^16^, ^19^ Hence, HCPs often resort to working with families as informal interpreters.^3^, ^20^ Though family members can provide additional medical histories about the patient, advocate on the patient's behalf and provide strong emotional support,^17^, ^21^ family members are ineffective interpreters. This is because their lack of medical training causes them to provide inaccurate translations and sometimes, they may even filter out information for personal agendas, thereby raising ethical concerns.^21^

Besides involving interpreters, HCPs can use digital tools (e.g., Google Translate) to translate simple medical information into the patient's mother tongue. Sole usage of digital tools often leads to clinical errors though, as HCPs are unable to fact‐check translated texts and digital tools often provide literal translations instead of culturally attuned translations.^22^, ^23^ Digital tools may also hinder meeting migrant patients' affective needs when HCPs neglect using non‐verbal cues (e.g., maintaining eye contact) due to technology usage during medical encounters.^24^ When ad‐hoc interpreters, informal interpreters and digital tools are used by default as substitutions for professional interpreters, this decreases the chance of migrant patients' cognitive and affective needs being met.^25^, ^26^ This translates into issues, such as the inability to navigate the healthcare system (e.g., knowing their rights) or feelings of alienation and discrimination.^27^, ^28^, ^29^, ^30^

To reach effective medical communication in LDCs, HCPs should work with interpreters and use digital tools in combination with *instrumental* and *affective communication strategies* advised by the *Six Function Model of Medical Communication* in a culturally and linguistically tailored manner. As there is a dearth of research on how HCPs currently use these communication strategies, this study aims to identify, among a heterogeneous group of HCPs, which strategies they use to bridge language barriers in LDCs. Results of this study may contribute to updating the current *Six Function Model of Medical Communication* in such a way that it can provide HCPs with an overview of the communication strategies they can use to meet specific communication goals they aim to achieve, thereby fulfilling migrant patients' cognitive and affective needs and promoting effective communication in LDCs. As a consequence, HCPs may receive better guidance on navigating LDCs and deter them from adopting the ‘getting‐by’ approach (i.e., relying on *some* communication strategies and underutilising professional interpreting services).^31^ This will help ensure migrant patients receive accurate medical information and are well‐engaged in their healthcare decisions. By emphasising the importance of using culturally sensitive communication approaches and highlighting the emotional impact of language barriers on migrant patients, the updated framework can also help HCPs to foster trusting patient–provider relationships. In sum, this study aims to answer the following research question:

*What communication strategies do HCPs report using to achieve the six goals of effective medical communication to meet the cognitive and affective needs of LLP migrant patients while mitigating language barriers during LDCs?*

## 2 METHODS

### 2.1 Study context

This study is part of a larger study, *Right2Health* (*Right2Health* is a consortium comprised of the University of Amsterdam, Catholic University of Leuven, Utrecht University and the University of Ghent). *Right2Health* will develop and evaluate an evidence‐based decision‐aid for helping HCPs and migrant patients to make shared decisions on the communication strategies they can use to mitigate language barriers and reach effective communication), funded by the Dutch Research Council. We employed a qualitative study design, and semi‐structured interviews were conducted with Dutch HCPs.

### 2.2 Recruitment and consent

The Amsterdam School of Communication Research (ASCoR) (2022‐PC‐14489) and the board of the Medical Ethical Committee of the Amsterdam UMC; location AMC; Amsterdam (W22_032 # 22.062) approved this study.

To generate a heterogeneous sample of HCPs, purposive and snowball sampling were used. Researchers (B. S., J. S., J. W.) contacted their respective networks of HCPs through email, where the researchers briefly stated what the project entailed. Those interested were then referred to the primary researcher (B. C.) who emailed them with an information letter and informed consent form. Interested participants were then forwarded to trained research assistants to schedule the interviews. Other participants were recruited via snowball sampling by asking participants about other HCPs who treat migrant patients and may be interested in participating in this study. These potential participants were then contacted the same way as with the purposive sampling method. All participants gave written informed consent.

### 2.3 Participants

The inclusion criteria were HCPs aged eighteen and above, based in the Netherlands and actively treating migrant patients at least twice monthly. This selection was guided by several factors. First, a scarcity of HCPs in the Netherlands exclusively devoted to treating migrant patients led to the dispersion of this group's admissions across healthcare institutions. Second, a prior study^32^ uncovered limited daily interactions between nurses, doctors and migrant patients. Finally, we determined that treating a couple of migrant patients monthly provided adequate exposure for HCPs to recall communication strategies used in LDCs, negating the necessity for a higher frequency to ensure reliable responses to interview questions. Therefore, the inclusion criterion was established for HCPs actively treating migrant patients at least bimonthly.

Twenty‐seven HCPs from different disciplines were interviewed (*M*_age_ = 45.07, SD = 11.46): general practitioners (*n* = 7), nurses (*n* = 3), mental HCPs (*n* = 4) and medical specialists (e.g., paediatricians, infectious disease specialists, etc.) (*n* = 13). Table 1 depicts participants' demographic information. The sample size of this study was determined based on theoretical saturation.^33^

**Table 1:** Background characteristics of participants.

Characteristic	*N* = 27	% Or mean
Gender		
Female	15	55.56
Male	12	44.44
Age (mean)		45.07 (SD = 11.46)
Religion		
Catholicism/Christianity	4	14.81
Judaism	1	3.70
Islam	2	7.41
None/prefer not to say	20	74.07
Country of birth		
The Netherlands	24	88.89
Other	3	11.11
Specialisation		
General practitioner	7	25.93
Specialist (internist)	13	48.15
Nurse	3	11.11
Mental health provider	4	14.81
Years in current practice		
0–5	5	18.52
6–10	6	22.22
11–15	5	18.52
16–20	3	11.11
21+	8	28.57

### 2.4 Data collection

Data collection took place between February 2022 to May 2022. None of the interviewers had prior relationships with the participants. Interviews were conducted independently by the first author (B. C.) and two trained research assistants. Seventeen interviews were conducted in English and ten in Dutch. All interviews were audiotaped and transcribed verbatim, and the Dutch transcripts were translated into English before analysis. B. C. provided background information and guidelines to the translator, who had previous experience with qualitative research to ensure reliability. Face‐to‐face interviews (*n* = 8) were conducted at the HCPs' office and online interviews (*n* = 19) were conducted via video call. On average, the interviews lasted an hour.

The topic guide for the interviews consisted of two parts and Table 2 shows an overview of questions reported in the current paper. The questions were developed using the *Six Function Model of Medical Communication* ^9^ and were revised based on discussions made with the entire *Right2Health* consortium.

**Table 2:** Part 1 of the semi‐structured interview topic guide.

*Meeting cognitive needs: Main questions*	
1	What do you do to collect medical information from your patient?
2	What do you do to provide accurate information about your patient's illness?
3	What do you do to check if they understood all information provided?
4	What do you to do understand the patient's treatment preference and advising them how to follow the treatment plan?
5	Do you think there are some communication methods that could have been useful, but you did not use? What do you think stopped you from using them?
*Meeting affective needs: Main questions*	
1	What do you do to meet the affective needs of the patient at the beginning of the consultation?
2	When you encounter a distressed patient, what do you do to resolve this?
3	What do you do to gain an understanding of the patient's values and beliefs that are related to their cultural background and/or religion?
4	What do you do to ensure that your patient feels supported after the consultation?
5	Do you think there are some communication methods that could have been useful, but you did not use? What do you think stopped you from using them?
*Additional probing questions for all main questions*	
1	Do you remember any specific cases where a professional or informal interpreter was present? Was there a difference in the communication methods you used?
2	Based on your experience, do you also think there are differences depending on: a. Patients' Dutch language proficiency? b. Health condition and treatment phase? c. Patient's sex and age? d. Patients' level of acculturation? e. Patients' health literacy?

### 2.5 Data analysis

All interviews were independently coded with ATLAS.ti version 22.0.2 by B. C. and one trained research assistant. Data were analysed employing the constant comparative method from Grounded Theory.^34^ Two out of twenty‐seven interviews were double‐coded independently, and interpretative codes were discussed until mutual agreement had been achieved. The first two interviews were subsequently recoded with the agreed codebook. No further revisions in the codebook were needed and the remaining interviews were coded and analysed deductively and inductively. Participants' experiences were first *deductively* coded by the interview questions (e.g., communication strategies used in meeting cognitive and affective needs) in the topic guide. The extracted data were then analysed *inductively* to categorise the codes further by grouping relevant responses and themes that emerged together within each topic guide question. Final pattern codes (i.e., key themes) were discussed among the research team until mutual agreement had been achieved.

## 3 RESULTS

### 3.1 Themes

**Figure 1:**
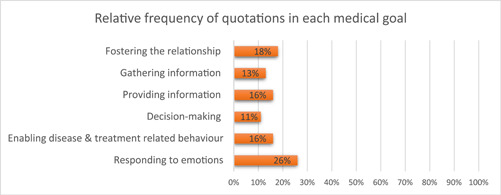
Relative frequency of quotations coded from all HCPs in each medical goal.

Five key themes emerged: (1) Getting‐by with instrumental communication strategies in dyadic and triadic LDCs; (2) Getting‐by with affective communication strategies in dyadic and triadic LDCs; (3) Using digital tools together with other communication strategies; (4) Working with various types of interpreters; (5) Involving additional support. HCPs spoke mostly about the communication strategies used to respond to emotions, and least about decision‐making (see Figure 1 for graph and Supporting Information S1: Appendix A).

**Figure 2:**
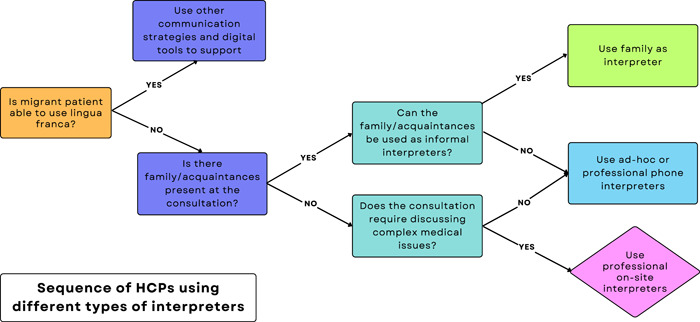
Healthcare providers (HCPs) used instrumental communication strategies and interpreters sequentially.

HCPs generally used communication strategies, digital tools and interpreters in a sequential manner (Figure 2). HCPs often used instrumental communication strategies (e.g., gestures and lingua franca) and digital tools (e.g., Google Translate) to communicate with migrant patients who visited the consultation alone. When HCPs could not communicate with patients and their Dutch‐speaking family members were present, HCPs would involve them as informal interpreters. If informal interpreters had low Dutch‐ or English‐language speaking proficiencies, HCPs would involve ad‐hoc or professional phone interpreters. Professional in‐person interpreters were only involved in consultations requiring the discussion of complex medical issues.

The key themes will be discussed under each medical goal from the *Six Function Model of Medical Communication* ^9^ (see Table 3 for an overview).

**Table 3:** Linkage between the six goals and five key themes emerged.

Key themes /goals		Fostering a relationship	Gathering information	Providing information	Decision‐making	Enabling disease and treatment‐related behaviours	Responding to emotions
1	Getting‐by with instrumental communication strategies		x	x	x	x	x
2	Getting‐by with affective communication strategies	x					x
3	Using digital tools together with other communication strategies^a^		x	x		x	x
4	Working with different types of interpreters	x	x	x	x	x	x
5	Involving additional support^a^		x	x		x	x

*Note*: This shows an overview of how the themes are related to the six goals in answer to our research question.

^a^ New strategies that emerged.

### 3.2 Fostering the relationship

At the beginning of the consultation, HCPs described using some affective communication strategies to foster a relationship with migrant patients. Most HCPs said they engaged in informal chats with existing and new migrant patients in dyadic and triadic LDCs. The informal chats included speaking in the patient's mother tongue and/or asking about their day. In dyadic LDCs with new migrant patients, HCPs used these chats as an assessment of whether or not to use a lingua franca:

Once you know that they cannot speak or understand Dutch, I would propose a different language like English, and see if both our levels of English are sufficient to do the conversation. (Female, 41, Internist)

HCPs attempted to increase migrant patients' sense of trust too with informal chats, such as via expressing empathy. HCPs also informed patients that they prioritised their comfort, by reinstating that the consultation room was a safe environment. A few HCPs also mentioned asking for migrant patients' expectations before gathering medical information:

[I] ask carefully what someone wants, […] how do you envision the treatment, what would you like? (Male, 50, Practice Assistant Mental Healthcare)

HCPs reported using non‐verbal affective communication strategies to build rapport and prioritise patients' comfort in dyadic and triadic LDCs. More than half of the HCPs said they emphasised maintaining eye contact to ‘search’ for contact and to express concern. A few mentioned that they would maintain less eye contact with female migrant patients compared to male migrant patients. Sometimes, maintaining open body language was mentioned. Most HCPs described expressing friendliness, leaning backwards or pointing towards the chair to invite migrant patients into the consultation space.

In LDCs where migrant patients were unable to communicate in a lingua franca, HCPs reported working with family members as informal interpreters frequently. However, before discussing medical concerns, HCPs indicated seeking migrant patients' consent to use family as interpreters first. HCPs indicated doing so to show respect and eliminate potential confidentiality issues. Most HCPs said they engaged in informal chats with family interpreters too. While doing so, some HCPs explicitly explained that they would maintain eye contact, mainly with the migrant patient:

I tell the daughter, OK, I'm gonna just look at your mom when I'm explaining things, even though I'm actually talking via you […] because I want to make the mother feel heard. (Female, 29, Practice Assistant Mental Healthcare)

When family interpreters were unavailable, HCPs would arrange either an ad‐hoc or professional phone interpreter. When HCPs said they used professional in‐person interpreters, they were usually arranged beforehand with existing migrant patients, or arranged for rescheduled appointments with new migrant patients.

### 3.3 Gathering information

After becoming acquainted, HCPs would move on to gather medical information from migrant patients. HCPs often adopted the ‘getting‐by’ approach, mainly getting‐by with lingua franca and gestures (i.e., asking migrant patients to point at body parts to indicate discomfort/pain) in LDCs to understand the patient's illness. Due to limited linguistic understanding by using lingua franca and gestures only, HCPs said they usually asked close‐ended questions instead of open‐ended questions. Open‐ended questions were only asked when migrant patients could adequately converse in a lingua franca.

When HCPs faced circumstances where using lingua franca and gestures insufficiently helped with communication, HCPs said they used digital tools, namely Google Translate. Sometimes, visuals were used, such as the digital visual analogue scale (for measuring migrant patients' acute and chronic pain levels) and Google Images for gathering information about migrant patients' physical wellbeing and emotional state. Only two HCPs mentioned using other digital tools (e.g., apps and patient portals) to gather information. One HCP said she would fill out an online questionnaire together with the migrant patient during the consultation to help the patient understand what was being asked:

If it's not possible, then I will just do it by hand and let them fill out [the questionnaire] in session. (Female, 29, Practice Assistant Mental Healthcare)

When professional and ad‐hoc interpreters were involved, HCPs described using instrumental communication strategies, such as asking questions, in a ‘normal’ way, that is, similar to language concordant consultations. However, professional in‐person interpreters were only used when HCPs needed to gather information about complex and difficult medical situations. When family interpreters were involved, HCPs mentioned avoiding asking relatively ‘private’ questions (e.g., about sexual health). If required, HCPs would reschedule the appointment:

Sometimes I say, listen, we need to [make] another appointment because [it is] not OK to discuss [this] with your family member here. (Female, 63, General Practitioner)

### 3.4 Providing information

After gaining an understanding of migrant patients' illnesses, HCPs moved on to providing information about their diagnoses. When HCPs could use lingua franca in dyadic LDCs, they continuously use it in combination with the following instrumental communication strategies: simplifying the information and providing information in small chunks. As providing simplified information in small chunks required more time than available in one consultation, almost all HCPs said they would schedule multiple consultations. More than half of HCPs also provided written information in Dutch or English to migrant patients who understood these languages. Few provided written information in migrant patients' mother tongue. For HCPs who did not provide written information, they noted most migrant patients had low reading literacy based on their personal experiences. To ensure that they could still get medical information across, these HCPs would use visuals instead—by making drawings to demonstrate the medical procedure.

HCPs frequently described asking migrant patients multiple times in dyadic LDCs for verification of understanding all medical information:

I ask, ‘Did you understand?’ I'll ask it two or three times if necessary. (Female, 64, Mental Healthcare Provider)

Few HCPs indicated using the teach‐back method (i.e., asking patients to make summaries of information provided) with patients who spoke a lingua franca. However, when asked about *how* the teach‐back method was used, HCPs only described asking migrant patients if they understood the information provided.

When lingua franca and gestures insufficiently helped with information exchange, HCPs mentioned using Google Translate to translate common languages (e.g., Turkish and Chinese), or translation websites for rarer languages (e.g., dialects). Both tools were used to translate short sentences when they faced time constraints and the inability to find interpreters specialising in languages of lesser/limited diffusions. If educational videos were available, few HCPs would provide links to such videos to their migrant patients.

When HCPs worked with different types of interpreters, they worked with them in combination with all the instrumental communication strategies mentioned above. The main difference though, was that visuals (e.g., drawings) were not used.

Additionally, when professional in‐person interpreters were involved, all HCPs described that they would provide medical information in the same way as they do in language‐concordant consultations. Ad‐hoc interpreters were also involved similarly. When informal interpreters were present, all HCPs still described simplifying, providing medical information in small chunks and asking for verifications on patients' understanding. Many HCPs said they would provide written information in English or Dutch for migrant patients' families acting as interpreters to take home.

### 3.5 Decision‐making

After exchanging information, the next step for HCPs is to engage patients in decision‐making. However, only a few HCPs who communicated with migrant patients in a lingua franca asked questions about patients' beliefs and values. HCPs who explored migrant patients' (spiritual) beliefs and values were those who treated complicated medical cases:

I do operations where they have tumors, it's very important [to know] what you want, what values and wishes you have. (Male, 59, Internist)

Some HCPs said they asked general questions to gain some knowledge about their patients' preferences, but often, these questions remain trivial:

What do you fear when you take this medication? Are there other ideas you have about your disease which make you uncomfortable taking the medication? (Female, 41, Internist)

When HCPs asked about how they came to a final treatment decision, most said they made joint decisions with migrant patients. A few said that they took the lead and made the decision for them. One HCP said that he left it to the migrant patient to make the decision.

When interpreters were involved, the instrumental communication strategies used differed depending on the type of interpreter. When professional phone interpreters were used, a few HCPs asked about migrant patients' backgrounds and beliefs. When professional in‐person interpreters were used, HCPs would ask questions about both topics. Similarly, when ad‐hoc interpreters were used, both topics were explored too. However, HCPs said they would ask more about the migrant patients' values instead of their personal backgrounds. On the contrary, when informal interpreters were used, only questions about migrant patients' beliefs were asked.

### 3.6 Enabling disease and treatment‐related behaviours

When enabling disease and treatment‐related behaviours, HCPs got‐by with the same instrumental communication strategies mentioned under gathering and providing information: lingua franca, simplifying information, providing information in small chunks, providing additional written information and asking questions to discuss, explain and check patients' understanding of the treatment process. However, in addition to those, they also often involved additional support—especially if migrant patients have visited the clinic alone. Most HCPs asked about their living situation and their social circles to explore who can support and promote their treatment‐related behaviours:

I explore what their social environment is. When relevant, ask them who do you have [as] your friend, do you have family, what kind of work do you do, whom do you meet, whom do you trust? (Male, 48, Internist)

When HCPs treated migrant patients who described having no available network, they often said they would involve additional support from nurses, professional caregivers and/or the migrant patients' general practitioners and neighbours after the consultations.

When professional phone interpreters were used, all HCPs said they enabled migrant patients' treatment‐related behaviours the same way as they would in dyadic LDCs. When professional in‐person and ad‐hoc interpreters were present, HCPs said that they would also focus on asking migrant patients about their living situation and social context. However, with family interpreters, all HCPs said they would ensure migrant patients' families understood the treatment by using the teach‐back method. To improve understanding, HCPs also provided written information in Dutch or English, depending on the language they have been communicating in, for family members to take home:

I let them read it back; sometimes I also give them a note, I write it down and I'll give the most important information on the paper. (Male, 33, Internist)

### 3.7 Responding to emotions

Through the consultation, HCPs should respond to migrant patients' emotions. In dyadic and triadic LDCs, HCPs described using silences and maintaining eye contact the most to comfort migrant patients experiencing negative emotions. Few HCPs mentioned paying attention to responses that were questions to gauge migrant patients' concerns. Additionally, only a few HCPs said that they would verbally ask their migrant patients about their emotions, as most HCPs said they used silence to provide migrant patients space to express their (negative) emotions and to observe their emotions. Similar to how HCPs maintain eye contact at the beginning of the consultation, HCPs also use it towards the end of the consultation to ‘search’ for emotional cues with migrant patients.

In consultations where HCPs felt the need to gauge migrant patients' emotional wellbeing, they expressed using Google Translate or translation websites to ask them about it, especially if they had predominantly been communicating with it. Some HCPs mentioned using Google Images to gauge migrant patients' emotional wellbeing. Such a strategy was often used with migrant patients who could not converse in a lingua franca.

HCPs expressed using the same set of affective communication strategies when different types of interpreters were involved; working with professional phone interpreters was the only difference. While almost all HCPs emphasised maintaining eye contact with the patient, some HCPs said they would avoid remaining silent, as that makes the consultation ‘awkward’ as the professional phone interpreter is unaware of the situation. Some HCPs also said that they would avoid asking migrant patients about their negative emotions explicitly with a professional phone interpreter. Instead, they focused on discussing remaining treatment concerns or explored migrant patients' social environment to see how they could cope with negative emotions.

When professional in‐person interpreters were involved, they would verbally respond to migrant patients' emotions just as they would in language‐concordant consultations. If HCPs detected an amicable relationship between the migrant patient and family member in the beginning, they would not explore the need for involving others as HCPs saw them as sufficient support.

## 4 DISCUSSION

The current study explored the communication strategies used by HCPs in LDCs to achieve the six goals of meeting the cognitive and affective needs of migrant patients while mitigating language barriers. HCPs predominantly used instrumental and affective communication strategies as suggested by the *Six Function Model of Medical Communication* with migrant patients, when there was a language barrier. They also relied on Google Translate and other translation websites, although other types of digital tools (e.g., digital question‐prompt lists) are available. Although free professional interpreting services are available in some healthcare settings in the Netherlands, HCPs made minimal use of them. They often saw it as a last resort, citing time constraints and the lack of immediately available interpreters as the main barriers.^15^ Therefore, although working with professional interpreters may be financially feasible, they are still underutilised; HCPs mainly rely on ‘getting‐by’ in both dyadic and triadic LDCs.^31^, ^35^, ^36^

Some possible explanations for why HCPs in LDCs often ‘got‐by’ may be their high perceived confidence in overcoming the language barrier, an overestimation of their own and migrant patients' language skills and the perceived simplicity of the topic of the consultation.^29^, ^37^, ^38^ Although it is unclear whether the HCPs in this study were aware of the Dutch guidelines for interpreting in healthcare,^39^ they implicitly followed them. That is, HCPs are less likely to use interpreting services if they think that the consultation will be straightforward (i.e., the conversation can remain at a superficial level) and believe that the (migrant) patient will be able to understand them without additional help. Some HCPs argued that when interpreters are present, conversations become indirect and this interferes with their ability to achieve certain goals, such as gathering information.^38^ They would, therefore resort to using instrumental communication strategies (e.g., minimal lingua franca), rather than using professional interpreters. However, the extent to which these communicative behaviours are useful in LDCs is questionable. When HCPs persistently repeat and rephrase medical information to migrant patients, patients often respond minimally, suggesting that migrant patients have little to no understanding.^4^, ^14^

Although this study showed that HCPs in LDCs were mainly ‘getting‐by,’ this study is able to extend the *Six Function Model of Medical Communication* with two key findings. First, digital tools, namely Google Translate and translation websites, were most commonly used. This is likely because it is free, easy to access and use and reduces the length of the consultation compared to using interpreting services.^40^ Another possible reason is that HCPs may perceive interpreters as unreliable due to previous negative experiences.^5^ In this study, HCPs described professional interpreters as being unprofessional because they provide poor‐quality interpretation and introduce new communication problems. Therefore, HCPs may opt for the more ‘convenient’ option—of using online translation tools. As the use of online translation tools is commonly observed in existing research,^40^ we propose to extend the current framework by adding the use of digital tools with instrumental communication strategies as an additional strategy to gather information, provide information, enable disease and treatment‐related behaviours and respond to emotions.

The second key finding that emerged was the reliance of HCPs on others to achieve the goals of gathering and providing information and enabling treatment and disease‐related behaviours. In dyadic LDCs, HCPs would seek additional support from migrant patients' general practitioners or mental health providers. In triadic LDCs, although HCPs expressed concerns about the language skills of informal interpreters, they still relied on informal interpreters to achieve these goals. This may be because HCPs are aware that some migrant patients have a high level of trust in family members.^41^ Therefore, HCPs often ensure that informal interpreters understand treatment plans, rather than ensuring that the migrant patient fully understands. When HCPs cannot rely on informal interpreters, they turn to migrant patients' GPs or try to explore their social networks, such as patients' neighbours. Therefore, we propose to add ‘involving additional support’ as an extension under ‘gathering and providing information,’ and ‘enabling disease‐ and treatment‐related behaviours’ in the framework.

Our final finding indicated that HCPs tended to focus on migrant patients' understanding by simplifying medical information in dyadic and triadic LDCs by asking close‐ended questions. HCPs often simply sought migrant patients' confirmation of understanding rather than using the teach‐back method (i.e., asking them to summarise the information provided), and almost none explored migrant patients' personal backgrounds or value systems to engage in shared decision‐making. These communicative behaviours suggest that HCPs do not engage in a true two‐way interaction, but instead treat the consultation as more of a technical task.^36^ This may be because HCPs assume that migrant patients would not understand the information or be able to explain their situation well. This lack of interaction raises ethical concerns, as the HCPs in this study were unable to provide evidence that their migrant patients would struggle to communicate. This means that HCPs treat migrant patients differently from nonmigrant patients. These differences in communication are considered to be a form of indirect discrimination, as they may impede access to and quality of healthcare for migrant populations.^6^, ^7^, ^8^ It is possible that these assumptions are being used as an (unconscious) rationalisation to keep the conversation at a superficial level and avoid in‐depth conversations, which can be tedious and slow. As a result, migrant patients may feel alienated, helpless, frustrated and discriminated against.^27^, ^28^, ^29^ This highlights the need to work with professional interpreters to ensure that both the cognitive and affective needs of migrant patients are met.

### 4.1 Study strengths, limitations and suggestions for further research

The results of our study have helped extend the *Six Function Model of Medical Communication* by identifying the communication strategies used by HCPs to mitigate language barriers in LDCs. By collecting qualitative, open data from HCPs of various specialisations, we have also gathered evidence on the variety of communication strategies that HCPs use to meet specific medical goals across different types of consultations. Simultaneously, our data also highlight that many communication strategies (e.g., digital tools and professional interpreters) are underutilised. Such findings can inform policymakers on updating existing guidelines for tackling language barriers. This will help shift HCPs' current approach—the ‘getting‐by’ approach—to an approach that will allow migrant populations to receive better standards of care and improve their health outcomes overall.

However, this study is not without limitations. First, the HCPs interviewed did not differentiate the communication strategies they used between different types of patients. They did not describe tailoring the choice of communication strategies to the specifics of the patient's cultural and linguistic background. This means that, although we were able to provide an overview of how HCPs deal with migrant patients in general, observations on how HCPs deal with language barriers arising from the variability of languages, such as languages with limited distribution and the difficulty of finding appropriate translation tools, are limited. Second, we attempted to explore potential differences in the communication strategies used based on migrant patients' sociodemographics. However, it was difficult for HCPs to recall all the nuances in retrospective interviews. Therefore, we subsequently dropped the probing questions. Finally, as our research question focused on uncovering which communication strategies were used in LDCs, this study lacked indepth observations on HCPs' reasoning for choosing certain communication strategies over others.

To address these limitations, future research should attempt to broaden the participant demographics and interview HCPs working in medical settings that treat migrants with asylum‐seeking or refugee backgrounds. In addition, conducting interviews with a diverse range of migrant populations will help to identify and verify the communication strategies that HCPs use to interact with them. Finally, we also suggest conducting observational analyses to systematically examine how HCPs use communication strategies in practice with different migrant patient groups. Such future studies will help highlight the specific communication challenges faced by different migrant groups, thereby identifying the many layers (e.g., indirect discrimination, power differentials, trust issues) that further complicate the communication.^42^ Doing so will enable the development of multi‐layered interventions to improve the quality of communication and healthcare for LLP migrant patients.

### 4.2 Practical implications

HCPs' getting‐by approach reflects that they do not tailor the communication strategies used in LDCs to the needs of migrant patients. As they cited time and lack of immediate availability of professional interpreters as the main barriers, this shows the choice of communication strategies used was driven by pragmatism. To ensure HCPs prioritise involving professional interpreters and are aware of the benefits of using multiple communication strategies together, more awareness about the positive impact of tailoring communication strategies according to the patient's cultural and linguistic background is needed. Guidelines should also be developed to equip HCPs with knowledge of what combinations of communication strategies are best used during different consultations, thereby reshaping their current communicative habits.

## 5 CONCLUSION

The choice of communication strategies used by HCPs in LDCs is largely driven by pragmatism, rather than ensuring that language barriers are mitigated as much as possible. They mainly ‘get‐by’ in LDCs, using communication strategies, digital tools and interpreters sequentially. Although digital tools and professional interpreters can be good solutions for reducing language barriers, both remain underused. Guidelines and interventions need to be developed to improve HCPs' existing knowledge and skills about the variety of communication strategies, digital tools and interpreters available. This will help HCPs identify optimal ways to use all available resources to overcome language barriers arising in different medical scenarios. This will also safeguard effective medical communication in LDCs, thereby bringing about a positive change in the quality of communication for LLP migrant patients and enhancing their quality of care.

## AUTHOR CONTRIBUTIONS

**Brittany M. C. Chan**: Methodology; formal analysis; investigation; writing—original draft; project administration. **Jeanine Suurmond**: Resources; writing—review & editing; supervision; funding acquisition. **Julia C. M. van Weert**: Resources; writing—review and editing; supervision. **Barbara C. Schouten**: Conceptualisation; resources; writing—review and editing; supervision; funding acquisition.

## CONFLICT OF INTEREST STATEMENT

The authors declare no conflicts of interest.

## Supporting information

Supporting information.

## Data Availability

The data that support the findings of this study are available on request from the corresponding author. The data are not publicly available due to privacy or ethical restrictions.

## References

[1] CBS.nl . StatLine—population; key figures. Accessed June 13, 2022. https://opendata.cbs.nl/#/CBS/en/dataset/37296eng/table?ts=1661937103193

[2] Jacobs EA , Diamond LC , eds. Providing health care in the context of language barriers: International perspectives. Multilingual Matters. Channel View Publications; 2017.

[3] Kale E , Syed HR . Language barriers and the use of interpreters in the public health services. A questionnaire‐based survey. Patient Educ Couns. 2010;81(2):187‐191. 10.1016/j.pec.2010.05.002 20542656

[4] Czapka EA , Gerwing J , Sagbakken M . (2018). Invisible rights: barriers and facilitators to access and use of interpreter services in health care settings by Polish migrants in Norway. Scand J Public Health. 2018;47(7):755‐764. 10.1177/1403494818807551 30345877

[5] Bischoff A , Denhaerynck K . What do language barriers cost? An exploratory study among asylum seekers in Switzerland. BMC Health Serv Res. 2010;10(1):248. 10.1186/1472-6963-10-248 20731818 PMC2939598

[6] Al Shamsi H , Almutairi AG , Al Mashrafi S , Al Kalbani T . Implications of language barriers for healthcare: a systematic review. Oman Med J. 2020;35(2):e122. 10.5001/omj.2020.40 32411417 PMC7201401

[7] Chiarenza A , Dauvrin M , Chiesa V , Baatout S , Verrept H . Supporting access to healthcare for refugees and migrants in European countries under particular migratory pressure. BMC Health Serv Res. 2019;19(1):513. 10.1186/s12913-019-4353-1 31337406 PMC6651950

[8] Lebano A , Hamed S , Bradby H , et al. Migrants' and refugees' health status and healthcare in Europe: a scoping literature review. BMC Public Health. 2020;20(1):1039. 10.1186/s12889-020-08749-8 32605605 PMC7329528

[9] De Haes H , Bensing J . Endpoints in medical communication research, proposing a framework of functions and outcomes. Patient Educ Couns. 2009;74(3):287‐294. 10.1016/j.pec.2008.12.006 19150197

[10] Ha Dinh TT , Bonner A , Clark R , Ramsbotham J , Hines S . The effectiveness of the teach‐back method on adherence and self‐management in health education for people with chronic disease: a systematic review. JBI Database System Rev Implement Rep. 2016;14(1):210‐247. 10.11124/jbisrir-2016-2296 26878928

[11] Montague E , Chen PY , Xu J , Chewning B , Barrett B . Nonverbal interpersonal interactions in clinical encounters and patient perceptions of empathy. J Particip Med. 2013;5:e33.

[12] Riess H , Kraft‐Todd G . E.M.P.A.T.H.Y.: a tool to enhance nonverbal communication between clinicians and their patients. Acad Med. 2014;89(8):1108‐1112. 10.1097/ACM.0000000000000287 24826853

[13] Wittink H , Oosterhaven J . Patient education and health literacy. Musculoskelet Sci Pract. 2018;38:120‐127. 10.1016/j.msksp.2018.06.004 30017902

[14] Landmark AMD , Svennevig J , Gerwing J , Gulbrandsen P . Patient involvement and language barriers: problems of agreement or understanding? Patient Educ Couns. 2017;100(6):1092‐1102. 10.1016/j.pec.2016.12.006 28065435

[15] Schouten BC , Cox A , Duran G , et al. Mitigating language and cultural barriers in healthcare communication: toward a holistic approach. Patient Educ Couns. 2020;103(12):2604‐2608. 10.1016/j.pec.2020.05.001 32423835

[16] Chang H , Hutchinson C , Gullick J . Pulled away: the experience of bilingual nurses as ad hoc interpreters in the emergency department. Ethn Health. 2021;26(7):1045‐1064. 10.1080/13557858.2019.1613518 31046427

[17] Hadziabdic E , Albin B , Heikkilä K , Hjelm K . Family members' experiences of the use of interpreters in healthcare. Prim Health Care Res Dev. 2014;15(2):156‐169. 10.1017/S1463423612000680 23402584

[18] Flores G . The impact of medical interpreter services on the quality of health care: a systematic review. Med Care Res Rev. 2005;62(3):255‐299. 10.1177/1077558705275416 15894705

[19] Karliner LS . Three critical steps to enhance delivery of language services in health care. In: Jacobs EA , Diamond LC , eds. Providing Health Care in the Context of Language Barriers: International P*erspectives* . Blue Ridge Summit: Multilingual Matters; 2017:20‐34.

[20] Meeuwesen L , Ani E , Cesaroni F , Jonathan Ross JE . Interpreting in health and social care: policies and interventions. In: Ingleby D , ed. Inequalities in Health Care for Migrants and Ethnic Minorities. Garant Publishers; 2012.

[21] Zendedel R , Schouten BC , van Weert JCM , van den Putte B . Informal interpreting in general practice: comparing the perspectives of general practitioners, migrant patients and family interpreters. Patient Educ Couns. 2016;99(6):981‐987. 10.1016/j.pec.2015.12.021 26792389

[22] Downie J , Dickson A . Unsound evaluations of medical machine translation risk patient health and confidentiality. JAMA Intern Med. 2019;179(7):1001. 10.1001/jamainternmed.2019.1856 31260014

[23] Turner AM , Choi YK , Dew K , et al. Evaluating the usefulness of translation technologies for emergency response communication: a scenario‐based study. JMIR Public Health Surveill. 2019;5(1):e11171. 10.2196/11171 30688652 PMC6369422

[24] Vieira LN , O'Hagan M , O'Sullivan C . Understanding the societal impacts of machine translation: a critical review of the literature on medical and legal use cases. Inform Commun Soc. 2021;24(11):1515‐1532. 10.1080/1369118X.2020.1776370

[25] Heath M , Hvass AMF , Wejse CM . Interpreter services and effect on healthcare—a systematic review of the impact of different types of interpreters on patient outcome. J Migr Health. 2023;7:100162. 10.1016/j.jmh.2023.100162 36816444 PMC9932446

[26] Panayiotou A , Gardner A , Williams S , et al. Language translation apps in health care settings: expert opinion. JMIR Mhealth Uhealth. 2019;7(4):e11316. 10.2196/11316 30964446 PMC6477569

[27] Rocque R , Leanza Y . A systematic review of patients' experiences in communicating with primary care physicians: intercultural encounters and a balance between vulnerability and integrity. PLoS One. 2015;10(10):0139577. 10.1371/journal.pone.0139577 PMC4594916 26440647

[28] Schinkel S , Schouten BC , Kerpiclik F , Van Den Putte B , Van Weert JCM . Perceptions of barriers to patient participation: are they due to language, culture, or discrimination? Health Commun. 2018;34(12):1469‐1481. 10.1080/10410236.2018.1500431 30040497

[29] Steinberg EM , Valenzuela‐Araujo D , Zickafoose JS , Kieffer E , DeCamp LR . The “battle” of managing language barriers in healthcare. Clin Pediatr. 2016;55(14):1318‐1327. 10.1177/0009922816629760 PMC4990509 26896341

[30] van den Broek T . Length of stay, acculturation and transnational medical travel among Polish migrants in the Netherlands. Int J Intercult Relat. 2021;84:210‐219. 10.1016/j.ijintrel.2021.08.002

[31] Diamond LC , Schenker Y , Curry L , Bradley EH , Fernandez A . Getting by: underuse of interpreters by resident physicians. J Gen Intern Med. 2008;24(2):256‐262. 10.1007/s11606-008-0875-7 19089503 PMC2628994

[32] Dias S , Gama A , Cargaleiro H , Martins MO . Health workers' attitudes toward immigrant patients: a cross‐sectional survey in primary health care services. Hum Resour Health. 2012;10:14. 10.1186/1478-4491-10-14 22776316 PMC3422994

[33] Francis JJ , Johnston M , Robertson C , et al. What is an adequate sample size? Operationalising data saturation for theory‐based interview studies. Psychol Health. 2010;25(10):1229‐1245. 10.1080/08870440903194015 20204937

[34] Charmaz K . Constructing Grounded Theory. SAGE; 2014.

[35] Hsieh E . Not just “getting by”: factors influencing providers' choice of interpreters. J Gen Intern Med. 2015;30(1):75‐82. 10.1007/s11606-014-3066-8 25338731 PMC4284281

[36] Jones L , Sheeran N , Pines R , Saunders B . How do health professionals decide whether an interpreter is needed for families in neonatal and pediatric units? Patient Educ Couns. 2019;102(9):1629‐1635. 10.1016/j.pec.2019.04.004 30981411

[37] Lion KC , Gritton J , Scannell J , et al. Patterns and predictors of professional interpreter use in the pediatric emergency department. Pediatrics. 2021;147(2):e20193312. 10.1542/peds.2019-3312 33468598 PMC7906072

[38] MacFarlane A , Huschke S , Pottie K , Hauck FR , Griswold K , Harris MF . Barriers to the use of trained interpreters in consultations with refugees in four resettlement countries: a qualitative analysis using normalisation process theory. BMC Fam Pract. 2020;21(1):259. 10.1186/s12875-020-01314-7 33278882 PMC7719256

[39] Pharos . Kwaliteitsnorm tolkgebruik bij anderstaligen in de zorg [Dutch quality standard on interpreter use]. 2014. Accessed December 22, 2022. https://www.pharos.nl/kennisbank/kwaliteitsnorm-tolkgebruik-bij-anderstaligen/

[40] Aelbrecht U‐V , Behrends M , Matthies HK , Jan Uvon . Usage of multilingual mobile translation applications in clinical settings. JMIR Mhealth Uhealth. 2013;1(1):e2268. 10.2196/mhealth.2268 PMC4114476 25100677

[41] Parial LL , Amoah PA , Chan K , Lai D , Leung A . Dementia literacy of racially minoritized people in a Chinese society: a qualitative study among South Asian migrants in Hong Kong. Ethn Health. 2023;28(5):757‐780. 10.1080/13557858.2022.2139818 36322705

[42] Schouten BC , Manthey L , Scarvaglieri C . Teaching intercultural communication skills in healthcare to improve care for culturally and linguistically diverse patients. Patient Educ Couns. 2023;115:107890. 10.1016/j.pec.2023.107890 37437511

