# Supplementary material for: Uncovering communication strategies used in language‐discordant consultations with people who are migrants: Qualitative interviews with healthcare providers
Source: Health Expect. 2023 Dec 25;27(1):e13949. doi: 10.1111/hex.13949 (PMC10750019; doi:10.1111/hex.13949)
Supplement: Supplementary file 1 — Supporting information. [file HEX-27-e13949-s001.docx]

**Appendix A**

After the completion of coding, we used ATLAS.ti to count the frequency of quotations coded in each medical goal. We then divided the frequency by the total number of codes to generate relative frequencies of HCPs’ discussion of each medical goal to generate *Figure 1*.

*Figure 1* shows the overall distribution of quotations across the six medical goals. Each bar represents the relative percentage of quotations attributed to a particular medical goal, showcasing the proportional contribution of each medical goal to the overall discourse. In other words, the percentages signify how much HCPs mentioned a specific medical goal in comparison to all the medical goals they talked about, allowing us to inspect the prevalence of specific topics within the broader context of the dataset. All coding researchers received prior training, coding guidelines, and had meetings regularly to discuss coding challenges and potential biases to minimise variations in coding interpretations.

**Figures**

Figure 1 Relative frequency of quotations coded from all HCPs in each medical goal
